# Needs, rationale, and outcomes of leadership education in neurosurgery

**DOI:** 10.1371/journal.pone.0318976

**Published:** 2025-02-28

**Authors:** Janissardhar Skulsampaopol, Sylvia Shitsama, Yu Ming, Ake Hansasuta, Michael D. Cusimano

**Affiliations:** 1 Division of Neurosurgery, Department of Surgery, University of Toronto, Ontario, Canada; 2 Department of Surgery, Faculty of Medicine, Ramathibodi Hospital, Mahidol University, Bangkok, Thailand; 3 School of Medicine, Jomo Kenyatta University of Agriculture and Technology, Nairobi, Kenya; UCSF: University of California San Francisco, UNITED STATES OF AMERICA

## Abstract

**Background:**

Surgeons are expected to lead teams/organizations to achieve optimal patient outcomes; however, few receive formal education in leadership. The goals of the study were to: 1) assess the unmet needs and gaps in leadership education for neurosurgeons and residents/fellows; 2) identify factors associated with availability of leadership education, access to leadership positions and the similarities**/**differences across geographic regions and institutional type; 3) describe the associations between gender and leadership; 4) determine the impact of leadership education.

**Methods:**

International survey of 657 neurosurgeons, residents/fellows. A series of univariate analysis and multivariate were conducted to assess the association between specific variables and leadership outcomes.

**Results:**

Almost half (48%) indicated that leadership education did not exist in their organization. This lack was more notable in non-academic centers (p < 0.001), among neurosurgeons with less than 5 years of work experience (p = 0.03), and respondents from South America (p = 0.02). Nearly two-thirds (61.1%) reported never having leadership training. Significantly fewer respondents in the age range 35–44 years old (p = 0.02), those working in the Middle East (p = 0.02), neurosurgeons with work experience less than 5 years (p = 0.004), working in non-academic center (p = 0.02) attended leadership training. In contrast to the differences seen across geographic regions and types of institutions, overall, the proportions of males and females having access to leadership training and being offered leadership positions were similar. Among participants, 87.1% of those with leadership training were offered leadership roles, compared to 65.5% of those without leadership training (p <  0.001). Additionally, participants with leadership training experienced a burnout rate of 29.2%, whereas those without leadership training had a higher rate of burnout of 40.5% (p =  0.02).

**Conclusions:**

There is a pressing need to develop educational opportunities for leadership in neurosurgery, especially for younger neurosurgeons, neurosurgeons working in non-academic centers, in countries and non-academic institutions where leadership education is less accessible. Leadership education is associated with increased numbers of neurosurgical leaders at all levels as well as reduced levels of burnout.

## Introduction

Leadership is “the process of creating constructive change” which involves mobilizing individuals and teams toward a desired future [1]. Physicians and surgeons need to be effective leaders to excel in their positions. In Canada, leadership has been enshrined in the CanMEDS competencies, which define the roles that all physicians need in order to provide excellent patient care [2]. According to the “Leader” role in the CanMEDS framework, all physicians are expected to demonstrate leadership by: 1) contributing to the enhancement of health care delivery within teams, organizations, and systems, such as fostering a culture of patient safety; 2) responsibly managing health care resources to ensure optimal patient care; 3) exhibiting leadership in professional practice, including driving improvements in health care services and outcomes; and 4) effectively managing aspects of the careers, such as financial planning and the allocation of health human resources within a practice [3]. Effective leadership is associated with better patient outcomes through improved team collaboration, reduced medical errors, and enhanced quality of care in high-stakes environments [4–9]. Moreover, effective physician leadership fosters an organizational culture that mitigates burnout [10,11]. However, leaders in healthcare organizations are often promoted based on career accomplishments or academic productivity, typically without a demonstrated competency in leadership. As a result, many become “accidental administrators” [12,13] which their leadership skills often being learned on the job, leading to avoidable mistakes that could have been prevented through adequate leadership training.

Leadership is not only vital for administrators in business or healthcare management but also plays a critical role in the effectiveness of both medical and surgical teams, highlighting the need for dedicated leadership development at all levels. A cluster randomized trial found that stroke patients cared for by staff who completed a multiphase team training program, including workshops on team dynamics, problem-solving, and utilizing performance feedback, demonstrated greater functional improvements than those treated by staff without such training [14]. A double-blind, randomized controlled trial conducted among obstetrics-gynecology and emergency medicine (EM) residents on leadership training demonstrated that residents who received leadership training showed a statistically significant improvement in leadership scores, progressing from the “average” to “good” range both immediately and at the 6-month follow-up. In contrast, the control group remained consistently in the “average” category throughout the study [15].

The literature underscores the critical need for leadership education across multiple disciplines. Surgeons frequently indicate that they lack formal training in leadership. A study of plastic surgeons showed the need for surgical leadership, given the critical relationship between leadership and the high stakes situations presented by their daily work [16]. Only 32% of surveyed cardiology trainees and young cardiology from 39 countries reported that they had leadership training [17]. Seventy percent of radiation oncology residents believed that leadership training during residency would be highly beneficial and emphasized the need for dedicated time within the residency program to focus on leadership development [18]. Neurosurgery is a high-stakes field where surgical complications can lead to significant patient morbidity or mortality. Additionally, neurosurgery often involves emergencies, such as trauma, which require timely intervention. A lack of effective leadership, alongside insufficient technical skills, is a significant contributor to medical errors [7,8]. Consequently, effective leadership could be essential for neurosurgeons to reduce surgical complications, optimize team workflows, and achieve better patient outcomes in this demanding setting. However, to date no study has explored neurosurgical education around the need for leadership training in neurosurgery, the factors that promote it, and the potential it could achieve.

Literature shows that female physicians across medical specialties are significantly less likely to be promoted to leadership roles compared to their male counterparts [19–22]. Women remain underrepresented in neurosurgery [23–26] and often face challenges in advancing within the field, including achieving successful practice or attaining leadership positions [27–30]. Inequitable access to leadership training and opportunities, influenced by gender and other factors, can further exacerbate disparities, strain team dynamics, and contribute to increased burnout in neurosurgery. Therefore, we considered it essential to examine these critical issues in the present study to help highlight the potential elements needed in strategies to address and mitigate potential inequities in leadership.

The goals of the following study were to:1) assess the unmet needs and gaps in leadership education for neurosurgeons and residents/fellows; 2) identify factors associated with availability of leadership education and access to leadership positions and the similarities or differences of leadership training in neurosurgery across geographic regions and institutional types; 3) describe the associations between gender and leadership and 4) determine the impact of leadership education.

## Methods

### Questionnaire

A comprehensive literature review to identify barriers and challenges facing neurosurgeons and their trainees became the basis for a questionnaire. The questions in the questionnaire were reviewed to confirm their relevance, and a definition of “leadership” was provided to ensure all questions were understandable and had adequate face validity [31]. The questionnaire was also initially pilot tested with a group of 10 neurosurgeons to review all the questions in terms of their validity. All items with concerns regarding the clarity of the questions and the survey flow were revised based on their feedback and the revised questions were confirmed with this pilot group before delivering the survey. The focus of this paper centers on leadership-related questions.

Questions covered a broad range of topics such as age, gender, level of training, work experience, work environment, position and geographic region of practice. A series of questions pertaining directly to leadership were also incorporated into the questionnaire. These included questions about: were leadership lectures, seminars, or courses provided to residents and staff by their university or organization and why these courses were provided; whether the respondent had attended any leadership courses or seminars and the reasons why they did or did not; whether they believed the leadership courses had an impact on their leadership style. Staff neurosurgeons were also asked whether they had been offered a leadership position and if so, what that position was (leadership position was defined as leadership role in organizational, e.g., division head, hospital director or academic leadership, e.g., Lecturer, Assistant Professor); and finally, whether they accepted or declined that position and why. The following validated definition of “**leadership**” was also provided in the questionnaire: the process of creating constructive change, involving mobilizing individuals/teams toward a desired future [1].

Burnout was measured using a validated single-item burnout measure rated on a scale from 1–5 [32–34]. The measure breakdown is as follows: 1 = *“I enjoy my work. I have no symptoms of burnout;”* 2 =  *“Occasionally I am under stress, and I don’t always have as much energy as I once did, but I don’t feel burned out;”* 3 =  *“I am definitely burning out and have one or more symptoms of burnout, such as physical and emotional exhaustion;”* 4 =  “*The symptoms of burnout that I’m experiencing won’t go away. I think about frustration at work a lot;”* and 5 =  *“I feel completely burned out and often wonder if I can go on. I am at the point where I may need some changes or may need to seek some sort of help.”* For the purpose of this study, respondents were considered to have burnout if they chose 3, 4 or 5.

### Survey

An electronic survey was developed using Survey Monkey®. Cluster sampling, a type of probability sampling [35], was used to ensure that responses included neurosurgeons and trainees from all geographic regions. The survey was sent to neurosurgeons and trainees in Asia, Africa, Australasia, Europe, North America, South America and Middle East using neurosurgical organizations’ group chats (e.g., the Royal College of Neurological Surgeons of Thailand (RCNST) LINE group chat, Continental Association of African Neurosurgeons (CAANS) WhatsApp group chat), other social media platforms (e.g., Facebook, LinkedIn) and publicly available email addresses during May 25, 2021–February 9, 2022. To ensure participants had received the survey and maximize responses, a reminder message was sent in the second, fourth and sixth week following the initial invitation. In addition, snowball sampling [36] was also used as the respondents were asked to voluntarily forward the survey to their colleagues and trainees including members of the European Association of Neurosurgical Society (EANS). We chose to conduct the survey using SurveyMonkey® and social media platforms because it is cost-effective and capable of collecting a large sample size from respondents worldwide, including areas that may not be physically accessible. Participation was entirely voluntary, and respondents could end their participation at any time. To participate in the survey, the participants needed to provide consent by clicking the “NEXT” button below the consent page, indicating they had read the form and agreed to take part in the study. Our study was approved by Research Ethics Board of Unity Health-St. Michael’s Hospital.

### Statistical analysis

Only those returned questionnaires that had complete responses to questions regarding the availability of leadership curriculum in the respondent’s university or organization were included in the analysis. Respondents’ age, gender, level of training (neurosurgical trainees, resident or fellow versus neurosurgeons) and region of practice were summarized descriptively. Among practicing neurosurgeons, their work setting (academic teaching or university affiliated hospital versus non-academic hospital), position, and work experience were also examined.

Descriptive statistics were documented for each question.

For questions regarding whether or not respondents were offered either academic or organizational leadership positions, some respondents indicated that they currently hold an academic position but responded that they had never been offered a leadership position. These responses were changed to reflect a positive response to the question of ever being offered a leadership position. However, because of the original response these respondents did not answer the follow-up question regarding accepting or declining the offer. This was treated as missing data.

A series of univariate analysis was conducted to assess the association between specific variables and leadership outcomes. Specifically, the relationship between age, gender, level of training, region of practice, work experience, work environment (academic versus non-academic hospital) was examined for their association with (a) reporting availability of leadership curriculum, (b) having attended a leadership course and (c) being offered a leadership position. A subgroup analysis of gender and the reported availability of leadership curriculum, having attended leadership training, and being offered a leadership position was also conducted. Finally, the relationship between having attended leadership training and being offered a leadership position and the association between having attended leadership courses and having burnout were examined. Multivariate logistic regression analysis was also performed to identify the independent relationships between potential predictor characteristics and the outcomes. The findings are presented as an odds ratio (ORs) as these outcomes are binary, with a corresponding 95% confidence interval (CI) provided to represent the range of plausible values for the ORs. A P-value of less than 0.05 was considered statistically significant. All analyses were conducted using *Stata* version 14.2 (Stata Corp).

## Results

Of 657 responses, 510 respondents (324 neurosurgeons and 186 trainees) provided complete data on all variables and were included in our analysis (completion rate 77.6%). Demographics of the respondents are shown in **Table 1**.

**Table 1:** Demographics of respondents (N =  510).

Variables	N	%
**Age (N = 510)**		
18–24 years old	2	0.4%
25–34 years old	175	34.3%
35–44 years old	202	39.6%
45–54 years old	89	17.5%
55–64 years old	30	5.9%
65–74 years old	9	1.8%
75 years old and older	1	0.2%
Prefer not to answer	2	0.4%
**Gender (N = 510)**		
Cis-Male	351	68.8%
Cis-Female	148	29%
Others (non-binary, gender fluid, not sure)	4	0.8%
Prefer not to answer	7	1.4%
**Level of training (N = 510)**		
**Neurosurgical trainee**	**186**	**36.5%**
**Gender**		
Cis-Male	114	61.3%
Cis-Female	69	37.1%
Others	3	1.6%
**Level of training**		
Resident	156	83.9%
Fellow	30	16.1%
**Neurosurgeon**	**324**	**63.5%**
Cis-Male	237	73.1%
Cis-Female	79	24.4%
Others	8	2.5%
**Work environment of practicing neurosurgeons (N = 324)**		
Academic teaching/university affiliated hospital	169	52.2%
Non-academic (public service, private practice)	155	47.8%
**Position of practicing neurosurgeons (N = 324)**		
Lecturer	43	13.3%
Assistant Professor	45	13.9%
Associate Professor	32	9.9%
Professor	21	6.5%
Course/Program director	22	6.8%
Department Chair	15	4.6%
Head of Division	27	8.3%
Retired	8	2.5%
Others	6	1.9%
**Work experience of practicing neurosurgeons (N = 323)**		
< 5 years	108	33.4%
5–10 years	88	27.2%
11–20 years	68	21.1%
> 20 years	55	17%
Prefer not to answer	4	1.2%
**Region (N = 407)**		
Asia	183	45.0%
Africa	71	17.4%
North America	63	15.5%
Europe	44	10.8%
Middle East	23	5.7%
South America	13	3.2%
Australia	10	2.5%

N =  Number of respondents.

**Table 2** presents the findings pertaining to the availability and participation in leadership education and leadership positions. Close to half of the respondents (48%) reported that the university or hospital they work at do not provide leadership lectures, seminars or courses to residents, Faculty or staff while approximately one-third (38%) indicated these types of courses are available. Neurosurgeons working in non academic hospitals were almost twice as likely to indicate they had no opportunities for leadership education than their counterparts working in academic hospitals (64.5% versus 36.7%, OR 3.14, 95% CI 1.94–5.07, p < 0.001). Fourteen percent of the respondents did not know if leadership courses were provided by their organizations. Among the respondents who stated that leadership courses are provided by their programs or hospitals, the reported main goals of those courses were to improve leadership (57.2%), improve teamwork (54.6%), and career progression (52.6%).

**Table 2:** Presence of leadership curricula and opportunities for leadership positions.

Does your university/organization provide leadership lectures, seminars, or courses for residents, faculty, and employees? (510)		
	N	N (%)
** Yes**	**194**	**194/510 (38.0%)**
** Yes, why does your program/employer provide these courses? (194)**		
** **Leadership	111	111/194 (57.2%)
** **Teamwork	106	106/194 (54.6%)
** **Career progression	102	102/194 (52.6%)
** **Communication	96	96/194 (49.5%)
** **Managerial skills	89	89/194 (45.9%)
** **Organizational culture	77	77/194 (39.7%)
** **Decision making	73	73/194 (37.6%)
** **Emotional intelligence	47	47/194 (24.2%)
** No**	**245**	**245/510 (48.0%)**
** I do not know**	**71**	**71/510 (14.0%)**
**Have you attended any leadership courses or seminars? (504)**		
** Yes**	**196**	**196/504 (38.9%)**
** Have these courses impacted your leadership style? (194)**		
** **Yes	134	134/194 (69.1%)
** **No	22	22/194 (11.3%)
** **Not sure	38	38/194 (19.6%)
** Why did you choose to attend the courses? (194)**		
** **For career progression	105	105/194 (54.1%)
** **Out of my own decision/desire	102	102/194 (52.6%)
** **It is a core part of my training/practice/organization	65	65/194 (33.5%)
** **For networking	63	63/194 (32.5%)
** No**	**308**	**308/504 (61.1%)**
** Why did you not attend the leadership courses? (choose all relevant answers) (308)**		
** **No leadership course available	135	135/308 (43.8%)
** **It is not a core requirement in our center/organization	132	132/308 (42.9%)
** **Lack of time	114	114/308 (37%)
** **Financial barriers	72	72/308 (23.4%)
** **Not interested	53	53/308 (17.2%)
**Have you ever been offered a leadership position? (318)**		
** Yes**	**235**	**235/318 (73.9%)**
** Did you “accept” or “decline” to take that position? (167)**		
** Accepted**	**158**	**158/167 (94.6%)**
** ** **Why did you accept the position? (158)**		
** **Personal interest	116	116/158 (73.4%)
** **Career progression	106	106/158 (67.1%)
** **Support from family/friends	28	28/158 (17.7%)
** **Financial incentives	17	17/158 (10.8%)
** Declined**	**9**	**9/167 (5.4%)**
** Why did you decline the position? (9)**		
** **I could not tolerate more responsibilities	5	5/9 (55.6%)
** **Young family/caring responsibilities	5	5/9 (55.6%)
** **Responsibilities were not matched by adequate financial compensation	2	2/9 (22.2%)
** **Other reasons	1	1/9 (11.1%)
** No**	**83**	**83/318 (26.1%)**

N =  Number of respondents.

Just over a third of all respondents (38.9%) (**Table 2**) had an opportunity to attend the leadership courses or seminars. Career progression was the main reason reported as to why respondents participated in these courses (54.1%) and the majority of the respondents who attended (69.1%) believed these courses had some impact on their leadership style.

Conversely, nearly two-thirds of all respondents (61.1%) had never attended any leadership courses. The most common reasons reported for not attending were that there were no courses offered to them (43.8%) or it was not a requirement for their training or organization (42.9%).

Nearly three-quarters of neurosurgeons (73.9%) were offered a leadership position at least once and nearly all (94.6%) accepted it. Among the 5.4% who declined the offer, the most common reasons given were that they could not take on additional responsibilities (55.6%) and they had responsibilities to take care their families (55.6%). All respondents aged 65 or older were given the opportunity to hold a leadership position at least once (see **Table 3).**

**Table 3:** Univariate associations between age, gender, level of training, work experience/work environment of neurosurgeons, and region of practice with the availability of leadership course, attendance at leadership courses and being offered a leadership position.

	Leadership course is available in their organization N (%)	P-value	Attended a leadership course N (%)	P-value	Being offered a leadership position N (%)	P-value
**Age**						
18–24 years old	1/2 (50%)	0.72	2/2 (100%)	0.08	0/0	–
25–34 years old	68/175 (38.9%)	0.78	57/172 (33.1%)	0.06	20/40 (50%)	0.0002* (OR 0.29, 95% CI 0.14-0.62)
35–44 years old	66/202 (32.7%)	0.04 (OR 0.68, 95% CI 0.46-1.01)	65/200 (32.5%)	0.02* (OR 0.64, 95% CI 0.43-0.94)	106/155 (68.4%)	0.03* (OR 0.57, 95%CI 0.33-0.98)
45–54 years old	37/89 (41.6%)	0.45	40/89 (44.9%)	0.20	75/85 (88.2%)	0.0004* (OR 3.42, 95% CI 1.63-7.83)
55–64 years old	15/30 (50%)	0.16	24/30 (80%)	<0.001* (OR 7.02, 95% CI 2.72-21.34)	24/28 (85.7%)	0.14
65–74 years old	5/9 (55.6%)	0.27	6/8 (75%)	0.03 (OR 4.83, 95% CI 0.85-49.27)	8/8 (100%)	0.09
75+ years old	1/1 (100%)	0.20	0/1 (0%)	0.42	1/1 (100%)	0.55
**Gender**						
Male	133/351 (37.9%)	0.92	134/348 (38.5%)	0.79	175/232 (75.4%)	0.32
Female	57/148 (38.5%)		59/146 (40.4%)		55/79 (69.6%)	
**Level of training**						
Neurosurgeon	122/324 (37.7%)	0.81	125/322 (38.8%)	0.97	NA	NA
Neurosurgical trainee	72/186 (38.7%)		71/182 (39%)			
**Work experience of neurosurgeons**						
< 5 years	32/108 (29.6%)	0.03* (OR 0.58, 95% CI 0.34-0.98)	30/108 (27.8%)	0.004* (OR 0.48, 95% CI 0.28-0.81)	67/107 (62.6%)	0.001* (OR 0.43, 95% CI 0.25-0.75)
5–10 years	29/88 (33%)	0.27	27/88 (30.7%)	0.06	63/88 (71.6%)	0.58
11–20 years	30/68 (44.1%)	0.22	29/66 (43.9%)	0.35	56/65 (86.2%)	0.01* (OR 2.59, 95% CI 1.12-7.27)
> 20 years	30/55 (54.5%)	0.005* (OR 2.30, (95% CI 1.22-4.32)	37/55 (67.3%)	<0.001* (OR 4.16, 95% CI 2.16-8.19)	46/53 (86.8%)	0.02* (OR 2.66, 95% CI 1.04-6.75)
**Work environment of neurosurgeons**						
Academic teaching/university affiliated hospital	82/169 (48.5%)	<0.001* (OR 2.71, 95% CI 1.65-4.46)	76/169 (45%)	0.02* (OR 1.73, 95% CI 1.07-2.81)	167/167 (100%)	<0.001
Non-academic hospital	40/155 (25.8%)		49/153 (32%)		68/151 (45%)	
**Region**						
Asia	68/183 (37.2%)	0.60	64/182 (35.2%)	0.10	83/121 (68.6%)	0.12
Africa	20/71 (28.2%)	0.05	33/71 (46.5%)	0.20	31/38 (81.6%)	0.21
North America	43/63 (68.3%)	<0.001* **(OR 4.3, 95%CI 2.36-8.14)**	30/63 (47.6%)	0.16	40/41 (97.6%)	0.0001* (OR 18.2, 95%CI 2.93-746.07)
Europe	12/44 (27.3%)	0.10	20/44 (45.5%)	0.41	20/34 (58.8%)	0.04 (OR 0.47, 95%CI 0.21-1.07)
Middle East	9/23 (39.1%)	0.96	4/23 (17.4%)	0.02* **(OR 0.30, 95%CI 0.07-0.94)**	6/11 (54.6%)	0.15
South America	1/13 (7.7%)	0.02* **(OR 0.13, 95%CI 0.002-0.88)**	3/13 (23.1%)	0.21	7/12 (58.3%)	0.23
Australia	4/10 (40%)	0.93	7/10 (70%)	0.05	7/8 (87.5%)	0.35

CI =  confidence interval; N =  Number; NA =  Not applicable; OR =  Odds ratio; * Statistically significant.

The association between age, gender, level of training, work experience/work environment of neurosurgeons, region of practice on the availability of leadership training, attended leadership training and being offered leadership position is presented in **Table 3**. Univariate analysis showed that neurosurgeons with less than 5 years of work experience (p = 0.03), and respondents from South America (p = 0.02) were less likely to report leadership education was available in their organizations. Less than one-third of respondents from South America (7.7%), Europe (27.3%) and Africa (28.2%) reported that a leadership program was available in their workplace. Conversely, the availability of leadership training in the workplace was reported by a greater number of neurosurgeons with more than 20 years of work experience (p = 0.005), those working in academic centers (p <  0.001) and respondents from North America (p < 0.001).

Respondents aged 35–44 years old (p = 0.02), working in the Middle East (p = 0.02), neurosurgeons with less than 5 years of work experience (p = 0.004) and working in a non-academic center (p = 0.02) had less opportunity to attend leadership training. Less than one-third of respondents from the Middle East (17.4%) and South America (23.1%) attended the leadership program. On the other hand, respondents in the 55-64 age range and neurosurgeons with more than 20-year work experience were more likely to have attended a leadership course at least once (OR 7.02, 95% CI 2.72–21.34, p <  0.001 and OR 4.16, 95% CI 2.16–8.19, p <  0.001, respectively). Respondents from Australia had the highest proportion of leadership training attendance (70%) compared to other geographic regions.

**Fig 1:**
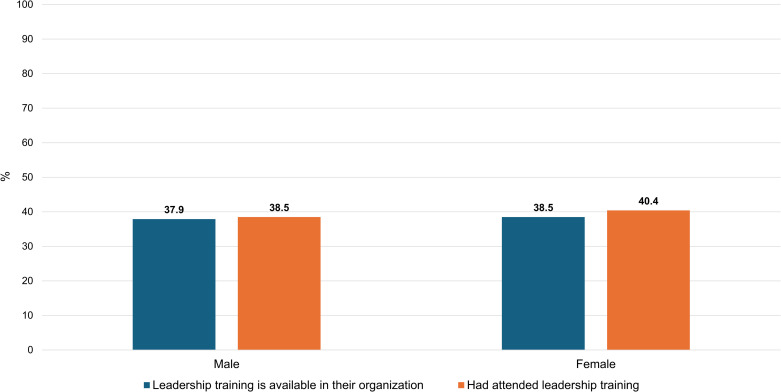
Differences in access to leadership education by gender.

**Fig 2:**
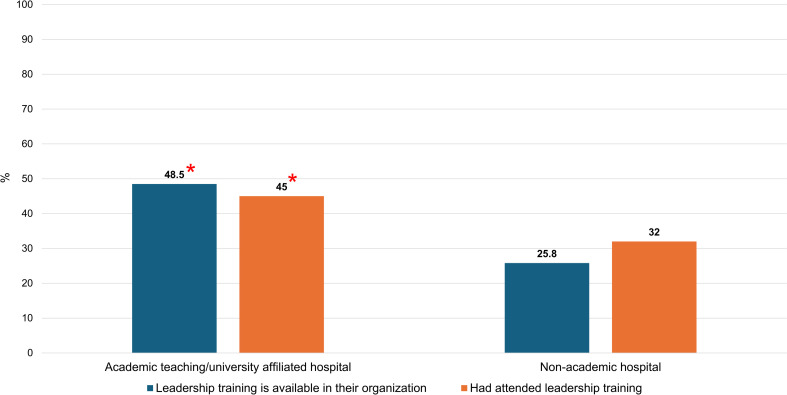
Differences in access to leadership education by institution. * Statistically significant.

**Fig 3:**
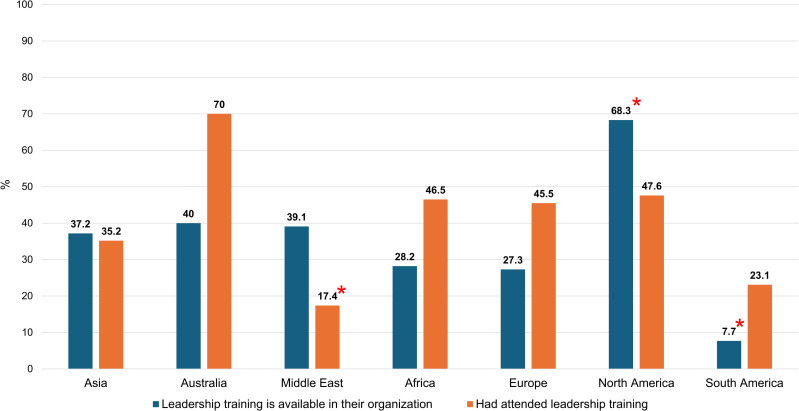
Differences in access to leadership education by geographic region. * Statistically significant.

**Figs 1**–**3** show differences in access to leadership education by gender, institution, and geographic region, respectively.

Neurosurgeons aged 45–54 years had the highest odds of being offered leadership positions in comparison to the other age groups (OR 3.42, 95% CI 1.63–7.83, p =  0.0004) while those in the 25–34 age category had the lowest odd of being offered leadership positions (OR 0.29, 95% CI 0.14–0.62, p =  0.0002) (Table 3). Neurosurgeons with 11–20 years of work experience, as well as those with 20 + years of experience were over two times as likely to be offered a leadership position (OR 2.59, 95% CI 1.12–7.27, p =  0.01 and OR 2.66, 95% CI 1.04–6.75, p =  0.02) respectively. On the other hand, neurosurgeons with less than 5-year work experience were much less likely to be offered leadership positions (OR 0.43, 95% CI 0.25–0.75, p =  0.001).

Respondents working in North America had the greatest odds of being offered leadership positions (OR 18.2, 95% CI 2.93–746.07, p = 0.0001) while neurosurgeons working in Europe were less likely to receive these offers (OR 0.47, 95% CI 0.21–1.07, p = 0.04). Table 3 shows that all neurosurgeons working in academic hospitals were offered leadership roles at least once, as the definition of “leadership position” in our questionnaire included academic leadership.

There were no differences in the likelihood of being offered leadership positions between male and female neurosurgeons.

**Table 4** focuses specifically on leadership positions offered to neurosurgeons. The top three positions reported were: Lecturer (46%), Head of Division (26.4%) and Director/Leader of Residency Education (17.5%). The leadership position offered least frequently was Full Professor (7.7%).

**Table 4:** Leadership positions offered to neurosurgeons (N =  235).

Position	N (%)
Lecturer	108/235 (46.0%)
Head of Division	62/235 (26.4%)
Director/Leader of Residency Education	41/235 (17.5%)
Leadership in a Professional Society (e.g., Neurosurgical Society)	39/235 (16.6%)
Leadership in sub-specialty Society	36/235 (15.3%)
Other University Leadership	27/235 (11.5%)
Chairperson of Department	26/235 (11.1%)
Associate Professor	26/235 (11.1%)
Hospital Director/Deputy Director	24/235 (10.2%)
Assistant Professor	24/235 (10.2%)
Full Professor	18/235 (7.7%)
Others	29/235 (12.3%)

N =  Number of respondents.

*Please note: one respondent may report multiple leadership positions.*

Subgroup analysis among neurosurgical trainees and neurosurgeons also supports the observation that there were no differences between males and females in terms of work environment, access to leadership education and leadership offered to neurosurgeons **Table 5**.

**Table 5:** Associations between gender and work environment, availability of leadership courses, attendance at leadership courses and being offered a leadership position among neurosurgical trainees and neurosurgeons.

	Academic teaching/university affiliated hospital N (%)	Reported that a leadership course is available in their organization N (%)	Attended a leadership course N (%)	Being offered a leadership position N (%)
**Neurosurgical trainee**				
Male	NA	42/114 (36.8%)	40/112 (35.7%)	NA
Female	NA	29/69 (42%)	30/67 (44.8%)	NA
P-Value	NA	0.51	0.25	NA
**Neurosurgeon**				
Male	124/232 (53.4%)	91/237 (38.4%)	94/236 (39.8%)	175/232 (75.4%)
Female	39/79 (49.4%)	28/79 (35.4%)	29/79 (36.7%)	55/79 (69.6%)
P-Value	0.52	0.65	0.54	0.32

N = Number; NA = Not applicable.

Multivariate analysis (see **Table 6**) showed that neurosurgeons from North America (p <  0.001) and neurosurgeons with work experience of more than 20 years (p = 0.008) were more likely to indicate that leadership training was provided by their organization. Neurosurgeons aged 55-64 years (p = 0.046) and those with more than 20 years of work experience (p = 0.041) were also more likely to attend leadership training at least once. The multivariate analysis revealed a strong association between leadership training (p = 0.003) and being offered leadership positions. In addition, respondents from North America (p = 0.042) were more likely to be offered leadership positions than respondents from other regions. In contrast, the odds of being offered leadership positions were significantly lower among neurosurgeons from Europe (p = 0.015) and those aged 25-34 years (p = 0.038) **Table 6**.

**Table 6:** Multivariate analysis predicting availability of leadership courses, attendance at leadership courses and experiences of being offered a leadership position.

Variables	OR	95% CI	P-Value
**Leadership course availability in the respondents’ organization**			
North America	4.27	1.94–9.42	<0.001*
Work experience > 20 years	2.71	1.30–5.68	0.008*
**Attendance at leadership courses**			
Age 55–64 years old	3.83	1.02–14.38	0.046*
Work experience > 20 years	2.44	1.04–5.74	0.041*
**Being offered a leadership position**			
Had attended leadership training	2.96	1.44–6.07	0.003*
North America	8.33	1.08–64.44	0.042*
Europe	0.31	0.12–0.79	0.015*
Age 25–34 years old	0.11	0.01–0.89	0.038*

CI =  Confidence interval; N =  Number; OR =  Odds ratio.

* Statistically significant

Table 7 shows that a statistically significant greater proportion of respondents who had attended leadership courses or seminars were offered a leadership position in comparison to those who had never attended such a course (87.1% versus 65.5%, OR 3.56, 95% CI 1.90–6.96, p <  0.001). Finally, respondents who had attended a leadership course were also significantly less likely to have burnout compared to those who had never attended such a course (29.2% versus 40.5%, OR 0.61, 95% CI 0.39-0.95, p =  0.02). **Table 7**.

**Table 7:** Associations between attending leadership courses with being offered a leadership position, and, with the presence or absence of burnout.

	Have attended leadership course N (%)	Have never attended leadership course N (%)	OR	95% CI	P-Value
**Being offered a leadership position**	108/124 (87.1%)	127/194 (65.5%)	3.56	1.90–6.96	<0.001*
**Presence of burnout**	47/161 (29.2%)	98/242 (40.5%)	0.61	0.39–0.95	0.02*

CI =  Confidence interval; N =  Number; OR =  Odds ratio.

* Statistically significant.

## Discussion

### The need for leadership education for neurosurgeons and neurosurgical trainees

Leadership is a critical skill essential for physicians to deliver optimal patient care and drive improvements in healthcare systems [2,4–6]. Our findings reveal that only slightly more than one-third of respondents have access to leadership training and have participated in leadership development events or faculty development within their organizations. This highlights a significant deficiency in leadership education across all levels of neurosurgery, from residents to faculty members, reflecting institutional neglect in this area. Moreover, slightly more than half of respondents cited career advancement or personal interest as their motivation for attending such training. This underscores a need for better communication about the broader benefits of leadership education, not just for professional growth but also for personal development in their roles as surgeons.

We found that an organization’s stance on supporting or neglecting leadership education significantly influences the fostering or inhibiting the development of neurosurgical leaders.

Merely offering leadership training opportunities is insufficient; ensuring trainee accessibility and participation is crucial for effective curriculum implementation [16,37]. Therefore, neurosurgical trainees and staff should be allocated protected time for leadership training, or course logistics should be adjusted to enhance participation rates. To promote greater involvement in leadership training, it should be easily accessible within or across organizations, supported by adequate protected time and financial resources to maximize its impact. The current shift towards online education presents an opportunity to deliver leadership training electronically, removing barriers related to travel, particularly beneficial for individuals with childcare responsibilities or lacking social support.

Our study revealed that the second most commonly cited reason for never attending leadership training was its lack of an organizational requirement or mandate. One potential solution is to implement a mandatory leadership curriculum in neurosurgical training programs. Given the essential role of leadership training in improving both administrative skills and overall patient care, there is a compelling case for universal access to and mandatory participation in leadership education, irrespective of gender, age, work experience, or professional setting. Major neurosurgical organizations certifying competence could introduce this as a mandatory component of training in the future.

To enhance leadership training availability, it will be important to consider the characteristics of successful training programs when developing and implementing such programs for neurosurgeons. Didactic sessions, mentorship, longitudinal study [1,12,13,37] and simulation [38,39] are each a viable approach. Organizations and programs considering implementing education in leadership would be wise to explore a broader body of knowledge regarding leadership education. Effective leadership involves guiding others toward challenging goals and requires the ability to analyze complex situations and devise strategic actions to achieve those goals [40]. Consequently, leadership development cannot be accomplished immediately after completing a training session; it demands continuous growth and lifelong learning [41–43]. As such, it is crucial to design longitudinal activities that transfer knowledge, skills, and attitudes from training to real-world applications [41–43]. A mentoring program is an effective method of leadership development. This involves pairing participants with a leader they admire and organizing monthly meetings to discuss case studies, reflect on how they handled them, and explore what could have been done better. Networking is another successful approach to leadership development, providing opportunities to learn from others’ experiences and mistakes related to leadership challenges [42,43]. A monthly departmental leadership seminar, conference, or roundtable discussion could focus on reviewing case studies and learning points from real-world scenarios, applying leadership principles to address issues such as delegating responsibilities and building trust with residents rotating through surgical units [40]. This initiative could serve as an engaging activity for lifelong leadership development while also offering practical solutions to common challenges faced by departmental administrators.

### Factors associated with leadership education and leadership positions

The results of our study revealed that more senior neurosurgeons were more likely to access leadership education and be offered leadership positions than their younger counterparts. These findings support the notion that many neurosurgeons in leadership positions are “accidental administrators” [12,13] as they have simply been offered a leadership position because their “time had come along.” It is clear that many in these positions have never received any formal leadership education or training to effectively lead their organizations. These coupled with the results presented here suggest that it is crucial for organizations worldwide to provide leadership education for all neurosurgeons and trainees and to prepare younger generations based on a gradual release of responsibility model akin to the development of technical skills [44–46]. We suggest that younger neurosurgeons should initially be assigned small-scale leadership roles to learn to lead, supported by mentors who facilitate their readiness for increased responsibilities over time. By gradually assuming more complex leadership roles and receiving formal leadership education, neurosurgeon leaders will be more likely to develop high competence in positively leading and influencing their organizations’ goals.

Our study revealed that neurosurgeons employed in non-academic hospitals had less access to leadership training opportunities and were less frequently offered leadership positions compared to their counterparts in academic settings. Additionally, neurosurgeons practicing in certain regions worldwide have fewer chances to access leadership education and opportunities for leadership roles. This underscores the critical need to develop leadership education, particularly for those with limited access, to enhance professional growth, organizational effectiveness, and improve patient outcomes in both academic and non-academic healthcare settings globally. Providing leadership education through neurosurgical organizations or societies, both locally and internationally, could effectively maximize access and address common challenges faced within neurosurgical communities, thereby enhancing practical application in their professional settings.

International neurosurgical organizations could consider organizing online leadership programs to expand access to leadership training in underserved countries. University-affiliated hospitals can provide leadership training to neurosurgeons working in nearby non-academic centers. Additionally, national neurosurgical organizations or medical associations should implement policies to ensure that leadership training is both mandatory and widely available.

### Gender and leadership in neurosurgery

Several prior studies indicate that female physicians, in general, are less likely to be promoted to leadership positions [19–22], a trend also observed specifically within neurosurgery [27–30].

However, our findings suggest that this trend might be evolving as no difference was observed in the likelihood of being offered leadership positions between cis-female and cis-male neurosurgeons in our study. Our study had too few respondents who did not identify as cis-male or cis-female to make any conclusions for those individuals not identifying as cis-genders. Although views on leadership responsibilities and gender roles may vary regionally and influence participation in leadership programs, literature shows that women in healthcare professions significantly lag behind their male counterparts in advancing into leadership roles. This disparity has prompted calls for gender equity, changes in traditional gender roles, and greater gender diversity in leadership across many regions of the world [47–50]. To reinforce this positive change, organizations should implement measures to ensure equal opportunities and support for all genders, along with developing policies to mitigate discrimination. Recognizing that mentorship is pivotal for career progression and academic success, we endorse prior calls that recommend formal mentorship and role models in leadership for young neurosurgeons of all genders to foster future leadership development [29,51,52].

### Impact of leadership curriculum on neurosurgeons and trainees

Our study revealed a significant association between participation in leadership courses and the likelihood of being offered leadership positions. This finding is consistent with previous research, such as the study on the American Academy of Orthopedic Surgeons (AAOS) Leadership Fellows Program, which showed a notable increase in the number of national committee chairs among orthopedic surgeons who had completed the program [53]. While it is possible that some individuals might attend leadership courses after being offered leadership positions, this scenario is less likely in our case. Our data showed that an equal proportion of those who attended leadership training and those who had not were offered leadership positions. Furthermore, among those who accepted leadership positions, approximately half had attended a leadership course and half had not. This balance suggests that participation in leadership training itself plays a crucial role in being offered leadership positions, rather than simply being a consequence of such offers.

The literature strongly shows that effective organizational leadership or supervisor leadership behaviors reduce burnout among physicians and healthcare providers [11,54–58]. One study found that a one-year leadership training program for head nurses significantly reduced burnout among the clinical nurses they supervised [59]. Our study is the first in healthcare profession to demonstrate that leadership training is directly associated with reduced burnout among the participants who engaged in the training themselves. This outcome is understandable given that leadership curricula for healthcare leaders often include the following components: (1) self-awareness (emotional awareness, recognition of one’s own leadership style); (2) self-management (achievement orientation, adaptability, emotional intelligence, time management); (3) social awareness (empathy, organizational awareness); (4) relationship management (negotiation, conflict resolution, teamwork, coaching/performance management, change management); and (5) other skills (leadership skills, financial skills, vision, values) [1,13,18,37 ]. Given the current and anticipated demands on physicians and the healthcare system in general, enhancing these core abilities and behaviors in physicians has the potential to not only develop leadership but also help reduce burnout at both individual and organizational levels.

It is also possible that our results reflect the established finding that organizations providing leadership education to their employees tend to foster a better organizational climate, which is a protective factor against burnout [11,37,54,55,59]. Additionally, individuals who have opportunities for leadership training often receive more social support from their organizations in general, and those with more support are less likely to experience burnout. Examining the impact of organizational climate and additional leadership support will be important concepts to address in subsequent research.

## Limitations

Our study has similar limitations to other cross-sectional surveys. It is limited to making statements of associations and cannot infer causality. As such, further study will be required to understand the causal relationships between the associations that we have uncovered. In addition, there may be a response bias. We had proportionately higher responses from female neurosurgeons and trainee respondents than the general neurosurgery population where approximately 8.2–12% are female staff [23–25] and 17.6% are female residents [26]. This may indicate that our results are biased toward the views of females who have traditionally been less represented in neurosurgery and in leadership positions. Furthermore, the results are limited to the representativeness of the respondents from each region of the world. The use of SurveyMonkey® and other digital platforms may limit participation among neurosurgeons with restricted internet access, particularly in certain geographic regions. To address this issue, future research could focus on regions with lower response rates and include alternative participation options, such as in-person surveys or telephone interviews, where feasible. These measures could help mitigate this limitation and improve response rates across all regions. In spite of these limitations, we had representation from every continent, the study is the largest of its kind to date in neurosurgery and provides valuable insights to help shape the future planning of education in neurosurgery and similar specialties in the future.

## Conclusions

Only 29.6% of neurosurgeons with less than 5 years of work experience and 7.7% of respondents from South America reported that leadership education was available in their organizations. In contrast, leadership training availability in the workplace was reported by 54.5% of neurosurgeons with more than 20 years of work experience, 48.5% of those working in academic centers, and 68.3% of respondents from North America. Furthermore, only 32.5% of respondents aged 35–44 years, 17.4% of those working in the Middle East, 27.8% of neurosurgeons with less than 5 years of work experience, and 32% of those working in non-academic centers had the opportunity to attend leadership training. Conversely, 80% of respondents aged 55–64, 67.3% of neurosurgeons with more than 20 years of work experience and 45% of neurosurgeons working in academic centers reported having attended a leadership course at least once. The study revealed that 87.1% of respondents with leadership training were offered leadership roles, compared to just 65.5% of those without such training (p <  0.001). Additionally, participants with leadership training experienced a lower burnout rate of 29.2%, compared to 40.5% among those without such training (p =  0.02).

Our novel study highlights the need for leadership education in neurosurgery and provides a rationale for guiding the development of education in the future. There is a pressing need to develop educational opportunities for leadership in neurosurgery, especially in younger neurosurgeons, neurosurgeons working in non-academic centers and in regions of the world where leadership education is less accessible. Such education should be supported for all and efforts encouraging gender equality regarding leadership training and opportunities should continue to be provided. Individual training programs and national certifying bodies have important opportunities to advance leadership education for future generations of neurosurgeons internationally. Future research could focus on the direct effects of leadership training on patient outcomes, participants’ burnout, organizational burnout, and the efficacy of structured leadership programs to identify what works best for neurosurgeons. Additionally, investigating the impact of mandated leadership training on neurosurgeons’ performance would provide valuable insights.
